# Enhancing Single-Mode Characteristics and Reducing Confinement Loss in Liquid-Core Anti-Resonant Fibers via Selective Filling and Geometrical Optimization

**DOI:** 10.3390/mi16040438

**Published:** 2025-04-05

**Authors:** Siyuan Chen, Caoyuan Wang, Cong Xiong, Yu Qin, Jie Zhu, Yichun Shen, Limin Xiao

**Affiliations:** 1 Advanced Fiber Devices and Systems Group, Key Laboratory of Micro and Nano Photonic Structures (MoE), Key Laboratory for Information Science of Electromagnetic Waves (MoE), Shanghai Engineering Research Center of Ultra-Precision Optical Manufacturing, School of Information Science and Technology, Fudan University, Shanghai 200433, China; 22210720006@m.fudan.edu.cn (S.C.); 18110720007@fudan.edu.cn (C.W.); 21110720019@m.fudan.edu.cn (C.X.); 22110720122@m.fudan.edu.cn (Y.Q.); 20110720112@fudan.edu.cn (J.Z.); 2 Zhongtian Technology Advanced Materials Co., Ltd., Nantong 226009, China; shenyc@ztt.cn

**Keywords:** anti-resonant liquid-core fiber, confinement loss, single-mode characteristics, finite element method, optical fiber optimization

## Abstract

The liquid-core anti-resonant fiber (LCARF) has emerged as a versatile platform for applications in nonlinear photonics, biological sensing, and other domains. In this study, a systematic and comprehensive analysis of LCARF was conducted via the finite element method to evaluate its performance across a wavelength range of 400–1200 nm. This included an assessment of the effects of structural parameters such as capillary wall thickness and the ratio of cladding tube diameter to core diameter on confinement loss and effective refractive index. The results reveal that the proposed core-only-filled approach significantly reduces the confinement loss compared to the conventional fully filled approach, thus facilitating signal transmission. Furthermore, the optimization of geometrical parameters greatly improves the single-mode characteristics of LCARFs. This work establishes a robust theoretical framework and provides valuable support for enhancing the LCARF applications in optofluidics, thereby contributing to the evolution of specialty fiber technologies.

## 1. Introduction

The interaction between light and liquid forms the cornerstone of numerous chemical and biological applications. For instance, in spectroscopy, absorption bands, or photoluminescence, enable the precise identification of liquid components, including suspended particles. However, the efficiency of spectroscopic investigations is generally constrained by the weak light–liquid interaction, which is caused by the high light energy loss, the limited interaction length, and limited overlap between the light field and the liquid region. Optical fibers, particularly those with capillary structures, have become promising platforms for the investigation of optofluidics due to the merits of low transmission losses and large light–liquid overlapping regions.

Since the 1970s, pioneers in optical fibers have explored the potential of liquid-filled fibers for various applications. Typically, high-refractive index (RI) liquid-core fibers, based on the transmission mechanism of total internal reflection (TIR), have been widely applicable in nonlinear optics [1,2,3]. However, in biological and chemical applications, low-RI liquids (e.g., water-based organic solutions, buffers, and cell culture media [4]) are far more common. The RI of these liquids is slightly higher than that of water (*n* = 1.33), while the RI of silica is approximately 1.47 in the visible and near-infrared (NIR) spectral ranges. Consequently, traditional fibers relying on TIR encounter difficulties in terms of guiding light in liquids with low RI.

Photonic-bandgap hollow-core fiber (PBG-HCF) and Kagome hollow-core fiber (K-HCF), which consist of tiny pores along the fiber length, have proved to be useful platforms for fiber-based optical fluid devices, implementing a completely different guidance mechanism to TIR. Despite their advantages, several limitations hinder their applications as liquid-core fibers, including the significantly constricted transmission bandwidth after media filling, challenges in fabricating small-core structures, and the difficulties of single mode transmission due to the existence of the surface mode. In recent years, anti-resonant fibers (ARFs) have attracted intensive interests from researchers [5,6,7]. Compared with traditional microstructure fibers, ARFs have more significant merits in transmission bandwidth and micropore size. For instance, the transmission band of ARFs does not disappear, even when filled with a liquid whose RI is close to that of the fiber material.

In addition, ARFs also have characteristics such as low-delay transmission, near-zero dispersion, and weak nonlinear response, which demonstrate great potential for applications in ultrafast pulse transmission and gas sensing [8]. Meanwhile, it should be noted that ARFs do not achieve 100% transmission efficiency due to incomplete reflection at the core–wall interface. Generally, low-order modes, with smaller incidence angles at the interface, experience higher reflectivity and lower losses, while the high-order mode has a larger incidence angle and higher loss. This inherent characteristic enables the cladding structure of ARFs to naturally filter high-order modes through resonant effects, thereby enhancing single-mode performance [9]. Moreover, ARFs filled with low-RI liquid also show their unique advantages in biochemical detection [10,11,12] and temperature sensing [13]. Therefore, conducting in-depth research on the transmission characteristics of liquid-filled ARFs is of extreme significance and can promote innovation and development throughout the entire field of specialty fiber technology, realizing a variety of functional applications.

This study comprehensively analyzed the influence of various key parameters on the transmission characteristics of liquid-core anti-resonant fibers (LCARFs). Specifically, we revealed the effects of the filling medium (unfilled, filled with ethanol, or filled with water) and filling method (fully filled or core-only filled) on the confinement losses and effective RI of different modes within the wavelength range of 400 to 1200 nm. On this basis, the single-mode characteristics of the designed LCARFs were explored. The optimal fiber structure for achieving single-mode transmission was determined via optimizing parameters including wall thickness and the ratio of the cladding tube diameter to the core diameter. The comprehensive and in-depth analyses conducted in this study can deepen our understanding with respect to the optical properties of the LCARFs, and lay a solid theoretical and data foundation for future experimental validation, design optimization, and potential applications.

## 2. Basic Model and Simulation Method

**Figure 1:**
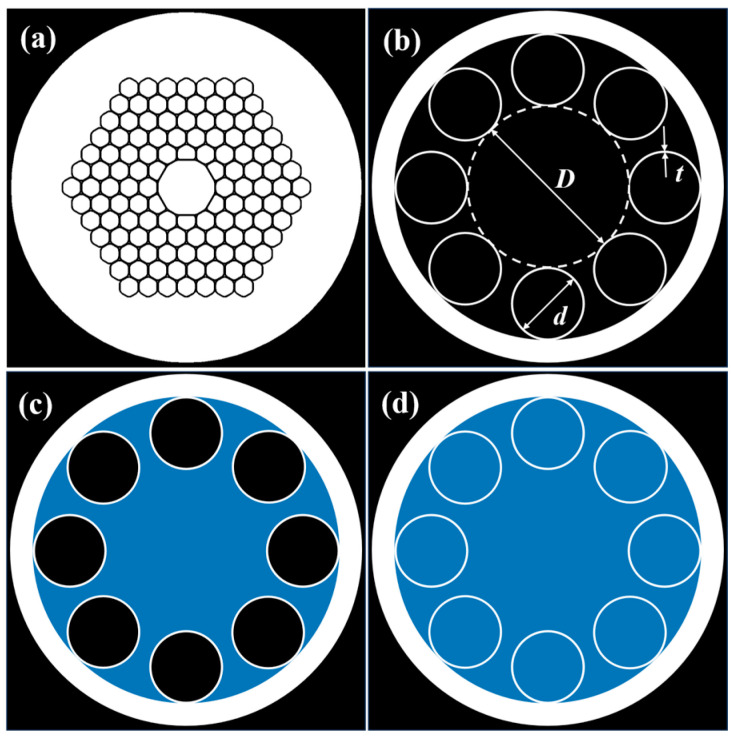
(**a**) A sketch of the PBG-HCF. (**b**) A sketch of the ARF with the key dimensions marked: core diameter D, capillary inner diameter d, and capillary thickness t. (**c**) A diagram of the structure, with liquid only filling the core area. (**d**) A diagram of the structure, with liquid filling the whole hollow area (the blue part is filled with liquid).

The majority of current studies on liquid-core fibers are based on the selective filling of PBG-HCFs or the full filling of ARFs, as shown in Figure 1a,b. In comparison with PBG-HCF, only ARF preserves the monolayer pores in the cladding zone, and the cladding tube wall is reduced to the size of a wavelength, ensuring the qualities of broad-band and low-loss transmission. Meanwhile, the issue of controlling the mode numbers in hollow-core fibers has been a focal point. PBG-HCFs have been plagued by the problem of excessive mode numbers for a long time, and the presence of a large quantity of higher-order modes (HOMs) has posed a significant challenge [14]. In the case of a large-core diameter, the confinement losses of HOMs in PBG-HCFs are generally on a par with those of the fundamental mode, which has a detrimental impact on the transmission performance. In practical optical transmission situations, multimode transmission typically leads to signal interference and increased attenuation. Although some degree of HOM suppression can be observed in small-core PBG-HCFs, other factors like surface roughness scattering make it extremely difficult to further reduce the attenuation to the level of traditional fibers. This clearly highlights the urgency and importance of resolving the issue of excessive mode numbers in HCF research. In contrast, ARFs present remarkable advantages in terms of mode control. Despite the fact that the hollow core of ARFs inherently supports multiple core modes, the nature of these modes’ interactions with the cladding is distinct. All modes, including the fundamental core mode, can undergo the leak of energy towards the cladding modes, even if the degrees of leakage are different. Significantly, the HOMs, due to the larger spatial extent in the dielectric cladding region, have a confinement loss that is two orders of magnitude higher than that of the fundamental mode [15]. Moreover, these HOMs have a greater tendency to resonate and couple with the cladding modes since the phase matching between the two sets of modes is relatively easier. Consequently, the HOMs quickly fade away within just a few meters of propagation while transmitting in the ARFs, effectively realizing few-mode transmission. This few-mode characteristic allows ARFs to significantly reduce signal interference and attenuation during optical transmission, thereby enhancing the stability and reliability of transmission. Essentially, ARFs provide a more advantageous solution for high-precision and low-loss optical transmission applications and present great application potential in the development of future optical communication technologies. Consequently, this work focused on the single-ring ARFs and explored their spectral characteristics when filling with some common liquids (water or ethanol) used in biochemical experiments. In addition, the influence of different filling methods (traditional full filling or core-only filling) on the transmission spectrum was investigated, as depicted in Figure 1c,d.

To accurately investigate the optical behavior of the ARFs, the finite element method (FEM) in COMSOL Multiphysics software (6.2) was employed for simulation purposes. Strict steps were followed to carry out the FEM simulation, and accurate structural parameters are prerequisites to ensure the effectiveness of simulation. Firstly, the geometric parameters of the fiber were obtained by scanning electron microscopy, and these are summarized in Table 1. Subsequently, the model was endowed with materials that were consistent with the actual physical properties of the fiber. In the definition of the boundary conditions, a cylindrical perfectly matched layer (PML) with a thickness of 5 μm was selected to ensure the efficient absorption and elimination of the light waves reflected back from the fiber boundary, thereby minimizing the potential interference of the boundary reflection. Given the robust confinement of the guided mode in the core, the set PML thickness was proven to be sufficient. According to the characteristics of different regions of the fiber structure, mesh elements of different sizes were adopted based on the fine meshing strategy. In addition, the mesh configuration which can ensure sufficient accuracy and effectively control the calculation cost was determined by comparing the simulation results at different mesh densities, and a detailed mesh convergence test was performed. The simulation of the LCARF was achieved by filling only the core region with water or ethanol. This setup enables the comparison of fiber transmission characteristics in the non-filled (full of air) and liquid-filled states. Meanwhile, the simulation was additionally performed when both the core and the cladding tube regions were filled with liquid.

## 3. Transmission Characteristics

The transmission mechanism of light in the ARFs can be explained by anti-resonant reflecting optical waveguide (ARROW) theory: the core of an ARF with a low-refractive index (RI) *n_core_* is surrounded by cladding with a high-RI *n_tube_*. The incident light is reflected by both the inner and outer surfaces of the ring-shaped cladding of the ARF, which acts as a Fabry–Perot resonator. When the resonant condition is met, the transmitted light intensity of the resonator is the largest and the reflection is the smallest, most of which leaks to the outside of the core. On the contrary, when the anti-resonant condition is met, the transmitted light intensity of the resonator is at a minimum while the reflected intensity is at a maximum. At this time, the optical waveguide is bound to be formed by reflection. For unfilled state, the resonant wavelength can be calculated according to equation [6]:

(1) $$\lambda_{m}=\frac{2t}{m}\sqrt{n_{tube}^{2}-n_{core}^{2}},m=1,2,3\ldots$$

where *t* is the thickness of the capillary wall. When the core is filled with low-RI *n_liquid_*, the optical environment in the fiber is changed, which affects the behavior of reflection, refraction, and interference. Specifically, under the condition of grazing incidence, the angle of incidence at the cladding wall–air interface exceeds the critical angle for TIR. This ensures that the TIR condition is met, preventing light from refracting out of the cladding wall into the air layer. Consequently, it becomes essential to refine the original theory by integrating relevant TIR principles.

When light is incident on the interface between liquid core and the cladding wall at a grazing angle of incidence *θ*_0_, a part of the light is reflected, and the remaining part enters the cladding wall. Then, TIR occurs at the interface between the cladding wall and air. A part of the totally reflected light refracts into the fiber core, while the other part is reflected again. In this way, multiple reflections and transmissions occur between the two interfaces, generating a large number of parallel light beams and forming multi-beam interference, similar to that in a Fabry–Perot resonator. However, at this time, due to the occurrence of TIR, the actual exit point of the reflected light has a small displacement parallel to the interface relative to that determined according to the laws of geometric optical reflection, that is, the Goos–Hänchen shift. Then, the resonant wavelength is as follows [16]:

(2) $$\lambda_{m}=\frac{4\pi n_{tube}t\cos\theta_{1}}{\phi +2m\pi }$$

where *θ*_1_ is the angle of refraction formed when light travels from the liquid core into the cladding wall. ϕ is the phase delay introduced by the Goos–Hänchen shift and can be calculated as follows [16]:

(3) $$\phi =\left\{\begin{array}{c} 2\tan^{-1}\left(\sqrt{\frac{2\Delta }{{cos}^{2}\theta_{1}}-1}\right),for\;s-polarization\\ 2\tan^{-1}\left(\frac{n_{1}^{2}}{n_{2}^{2}}\sqrt{\frac{2\Delta }{{cos}^{2}\theta_{1}}-1}\right),for\;p-polarization \end{array}\right.$$

where $\Delta =\frac{n_{1}^{2}{-n}_{2}^{2}}{2n_{1}^{2}}$.

The resonant phenomenon at this time can be explained from the perspective of modes. In the process of light transmission, the input light will experience a loss phenomenon under specific cladding wall thickness conditions when the wavelength or the input angle is constated. This phenomenon fundamentally stems from the coupling between the guided modes in the fiber core and the lossy modes in the cladding tube. When the lossy modes approach the cut-off state, the optical loss caused by this coupling will occur. Moreover, there exists a specific cut-off thickness for the cladding wall, at which the transmission loss reaches its maximum value. In addition, changes in the wavelength of the input light can also trigger similar phenomena. When the thickness of the cladding wall remains constant, a corresponding resonant peak will appear in the spectrum if a certain mode in the cladding wall approaches the cut-off state at a specific input wavelength. This kind of resonant peak is called a lossy-mode resonance [17].

### 3.1. Confinement Loss and Effective-Mode RI of HE_11_ Mode

The HE_11_ mode is the basic propagation mode in LCARFs and performs a pivotal role. Especially in scenarios requiring high sensitivity, dynamic adjustment, and long-distance stable communication, the HE_11_ mode in LCARFs demonstrates immense application potential and scientific research value. Therefore, the propagation characteristics of HE_11_ mode were focused on first.

**Figure 2:**
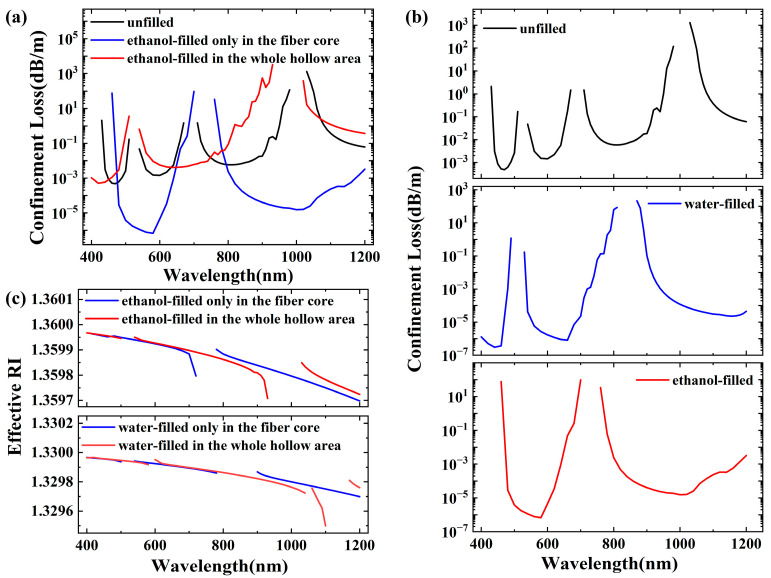
(**a**,**b**) Confinement losses of the fundamental core mode under different filling conditions; (**c**) the effective-mode RI of the fundamental core mode under different filling conditions.

Figure 2a,b show the confinement losses of the HE_11_ mode under different filling conditions within the wavelength range of 400–1200 nm, which aligns with the typical requirements for biomedical measurements and nonlinear phenomena. As shown in Figure 2a, when the whole hollow area of the fiber is fully filled with ethanol, the order of magnitude of the confinement loss remains relatively unchanged compared to its unfilled state. The blue shift in the transmission window is also notable, keeping the loss to less than 0.1 dB/m over 190 nm (third transmission window for unfilled state, 725 nm to 915 nm) and 250 nm (second transmission window for the state filled with ethanol in the whole hollow area state, 550 nm to 800 nm), respectively. When only the core area is filled with ethanol, the order of magnitude of the confinement loss is significantly reduced, with a loss of less than 0.001 dB/m over 165 nm (second transmission window, 475 nm to 640 nm). This is because the RI of the filling liquid changes the RI distribution pattern of the fiber. In the unfilled state, the optical properties of the fiber are based on the inherent RI of the core and cladding, and the specific waveguide structure and resonant conditions are formed. As discussed above, when the fiber core is filled with liquid, the effective RI is changed, affecting the light propagation characteristics. This change also has an impact on the propagation constant of light in the optical fiber. In view of the resonant condition, the change in the propagation constant will inevitably cause a corresponding change in the resonant wavelength meeting the resonant condition, resulting in the resonant wavelength deviation, and then lead to drift in the transmission window.

From Figure 2b, it can be seen that as the RI of the filling medium increases, the blue-shift phenomenon becomes more pronounced, which aligns with the theoretical analysis. Notably, at certain window edges, the loss curve no longer remains smooth but exhibits fluctuations as the wavelength changes, especially for the situation where the fiber core is filled with water. This fluctuation arises from the coupling between the core modes and the cladding modes. In the ARF with seven cladding capillaries, described in reference [18], a similar fluctuation in the loss curve is also observed at the right edge of the second-order transmission window, further validating our calculation results. Regarding the effective-mode RI, its accurate calculation is pivotal in designing high-speed, large-capacity communication systems. It aids in determining the transmission mode and dispersion characteristics of the optical signal within the fiber, enabling the selection of suitable light sources, modulation techniques, and receiving devices to enhance communication system performance. As shown in Figure 2c, it can be observed that different filling methods exert a clear drifting effect on its variation range. Specifically, the RI value remains relatively stable in the short-wavelength range but undergoes more significant changes in the long-wavelength range.

**Figure 3:**
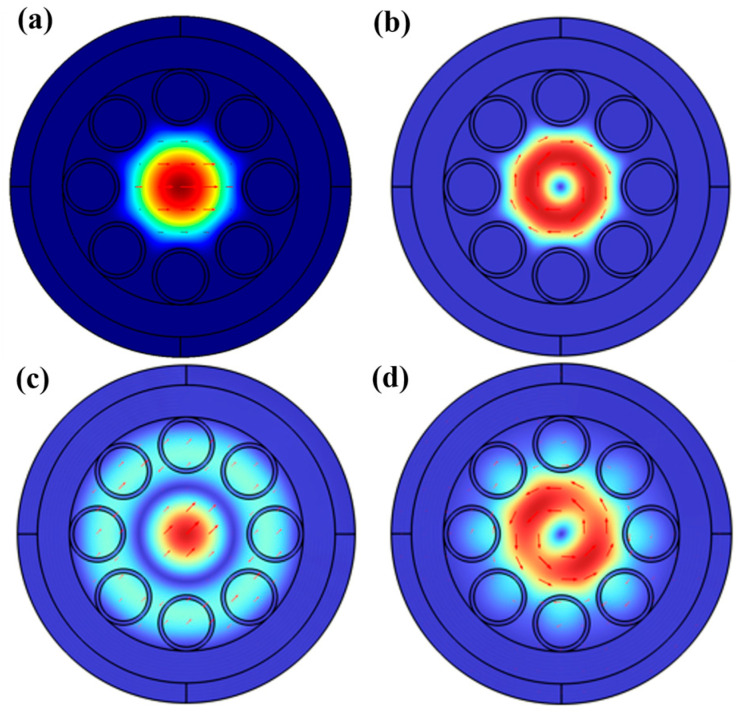
Modal intensity distributions of (**a**) HE_11_ and (**b**) TE_01_ core modes with ethanol only in the core and electric field distributions of (**c**) HE_11_ and (**d**) TE_01_ core modes with ethanol filled in the whole hollow area.

The anti-resonant reflection mechanism is crucial for impeding the penetration of light into the silica surrounding the core, which is essential for achieving the low loss characteristics observed in ARFs. However, this mechanism is not always fully effective. More generally, the core mode tends to couple with the cladding mode, leading to the loss and leakage of the core mode. When the anti-resonant reflection condition is met, there are not only effective modes in the fiber core, but there are also modes in the ring structure formed by the surrounding cladding tube. When the real part of the effective-mode RI of the core mode and the cladding mode is close, coupling occurs between the modes. If the core mode is coupled with the cladding tube mode, the loss of the core mode will significantly increase. Therefore, suppressing this coupling is crucial for the practical application of ARFs. To suppress the coupling, the leakage of the core mode to the cladding needs to be minimized. This can be realized by drastically reducing the interaction between the core mode and the cladding mode. This reduction can be achieved through a small spatial-mode overlap and a mismatch in wave numbers or effective-mode RI. For different filling methods, filling solely the fiber core can significantly reduce the transmission loss, approximately by a factor of 1000, increasing the depth of the transmission window. The notable increase in the depth of the transmission window implies its potential application in higher-performance bandwidth filters. This significant reduction occurs from the large difference in RI between the core mode and the cladding mode, which greatly increases the degree of inhibition of mode coupling and thus reduces the transmission loss. In other words, when the cladding tube remains unfilled, the optical field is better confined within the core, thereby minimizing the interference and leakage of light. This phenomenon can be more obvious when studied from the mode field, as depicted in the Figure 3, which illustrates the electric field distributions of HE_11_ and TE_01_ modes under different filling conditions. As shown in Figure 3a,b, both the fundamental mode and second-order mode are confined strictly within the fiber core when it is filled with ethanol only. Part of energy leaks into cladding tubes when the whole hollow area is filled with ethanol, as shown in Figure 3c,d.

Furthermore, this study provides an in-depth investigation of the TIR phenomenon induced by liquid-core filling. By continuously reducing the capillary wall thickness t, a distinct transition from the ARROW theory model into a TIR-dominated regime was observed. This transition holds significant implications for understanding light confinement mechanisms in LCARFs.

**Figure 4:**
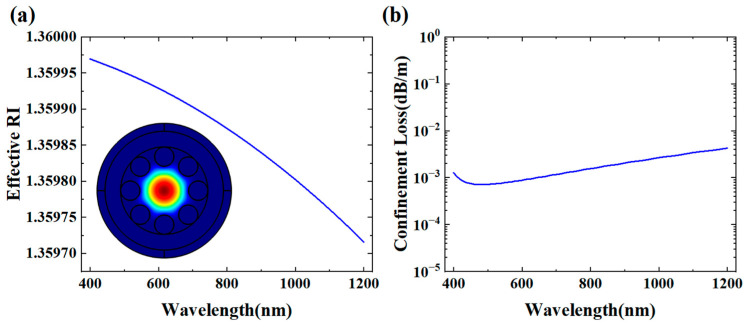
(**a**) Confinement loss and (**b**) effective-mode RI of fundamental core mode when core is filled with ethanol (t = 100 nm, D = 30.5 μm, d = 12 μm). Insert picture is electric field distribution.

When capillary wall thickness t was extremely small, the optical field could no longer establish effective Fabry–Pérot resonance within the wall, thereby completely suppressing the leaky-mode resonance. To verify the dominance of the TIR mechanism, we conducted detailed analysis of optical characteristics under conditions where the wall thickness t was set to be 100 nm when the core was filled with ethanol. As shown in Figure 4, both the confinement loss and the effective RI showed continuous variation without resonance phenomena across the entire studied wavelength range (400–1200 nm). The insert picture in Figure 4 further confirms the complete light confinement within the core region from the perspective of the mode field distribution.

### 3.2. Confinement Loss and Effective-Mode RI of HE_21_, TE_01_, and TM_01_ Modes

**Figure 5:**
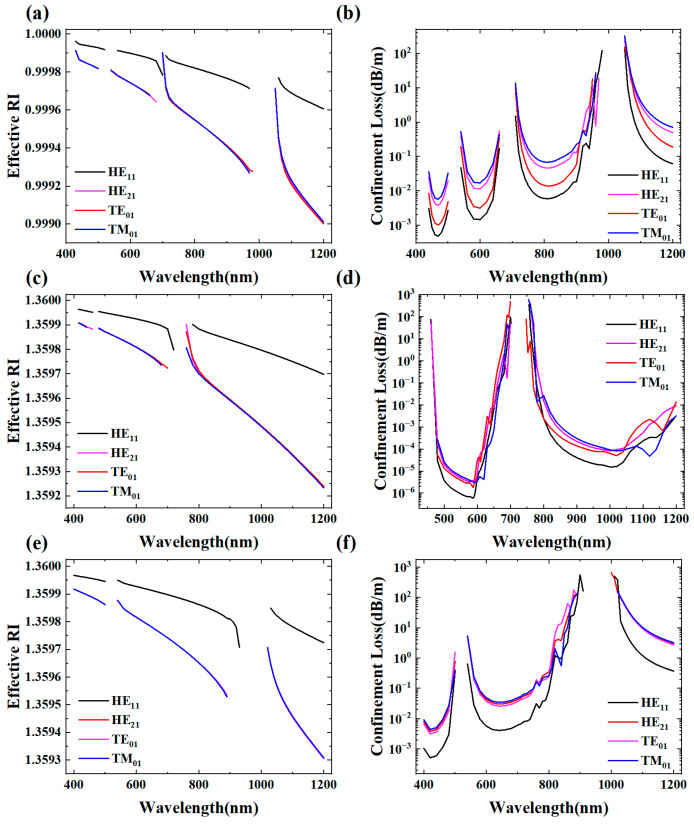
Mode effective RI and confinement losses of different core modes under different filling conditions: (**a**,**b**) unfilled; (**c**,**d**) ethanol-filled core; (**e**,**f**) ethanol-filled whole hollow area.

Figure 5 depicts the effective RI and confinement losses of different core modes under different filling conditions. As illustrated in Figure 5a,c,e, the three HOMs exhibit lower effective RI compared to the HE_11_ mode. Additionally, the effective RI of different modes of LCARFs shows a decreasing trend with the increase in wavelength. While the difference in effective RI between modes of the same order is relatively small, this difference exhibits slight augmentation as the wavelength continues to increase. Furthermore, modes of different orders exhibit varying degrees of change in their effective RI with changes in wavelength. From the perspective of the effective RI difference, this difference is magnified with an increase in wavelength. As shown in Figure 5b,d,f, the confinement losses of the three HOMs are always higher than those of the HE_11_ mode.

## 4. Structural Optimization

In the previous section, we demonstrated that our proposed liquid-filled core-only method is superior to the traditional all-filled method. In this section, the influence of fiber structure parameters on fiber transmission performance is further discussed on the basis of the model where ethanol only fills the fiber core.

**Figure 6:**
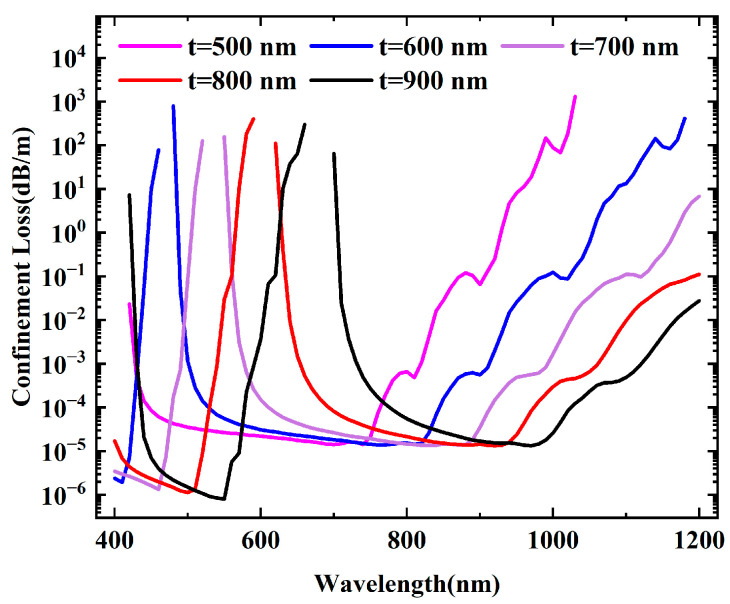
Confinement losses of the fundamental core mode at different capillary thicknesses.

Optimizing the performance of ARF by adjusting the sizes, shapes, and numbers of the cladding tubes is a flexible and effective strategy. Equation (2) shows that the alterations in capillary thickness have an important impact on the drift of the resonant wavelength, thereby directly determining the position of the transmission window. Therefore, the wall thickness of the cladding tube was studied first. The optimal capillary thickness can be ascertained by computing the fundamental mode confinement loss across different capillary thicknesses. Figure 6 illustrates the confinement loss variation as the capillary thickness ranges from 500 nm to 900 nm at an interval of 100 nm. As t gradually escalates from 500 nm to 900 nm, the corresponding resonant wavelength shifts from 400 nm to approximately 700 nm. This phenomenon stems from the modification of the effective RI difference between the annular channel and the core due to increased capillary thickness, which consequently alters the resonance conditions of light within the annular channel. When the capillary thickness increases, the RI difference between the core and the cladding tubes is relatively diminished (owing to a higher proportion of high-RI material in the wall), resulting in a change in the effective RI of light in the annular channel. This alteration modifies the phase accumulation of light as it propagates through the channel, necessitating a longer wavelength to fulfill the resonant condition, hence leading to the redshift of the resonant wavelength. According to the simulation, when t = 500 nm, the transmission window encompasses 532 nm and 633 nm, offering broader application prospects.

While ARFs demonstrate the ability to achieve few-mode transmission, there are still hurdles in maintaining pure low-order modes. Even if the pure LP_01_ mode is successfully obtained, the HOMs can be excited by fiber bending or stretching, resulting in the degradation of the transmission beam profile and power fluctuations. Therefore, in the development of ARFs, ensuring stable single-mode transmission remains a crucial and challenging task. This issue becomes particularly prominent in applications involving relatively short fiber lengths, such as in laser processing and pulse compression. As a result, extensive research efforts have been dedicated to investigating the single-mode characteristics of LCARFs in an attempt to overcome these challenges. Consequently, the single-mode characteristics of LCARF is then investigated.

Although an 8-ring ARF seems to have great advantages in low-loss transmission, this structure was proved to be unable to provide effectively single-mode operation [19]. On the contrast, the six-ring structure not only excels in inhibiting the coupling between the fundamental core mode and the cladding modes [20] but also shows better performance than the eight-ring structure in other respects. On the one hand, compared with the eight-ring structure, the six-ring structure may be more mature and simpler to use in the production process, thereby reducing the production cost and improving production efficiency, which makes the use of the six-ring structure more feasible in industrial production. On the other hand, under the premise of ensuring a certain performance, the stability of the six-ring structure may be better since it may be able to better maintain its optical performance in the face of environmental factors such as mechanical stress and temperature changes and reduce the performance decline caused by structural instability. To sum up, based on the production process, structural stability, and other considerations, the use of a six-ring structure instead of an eight-ring structure is a reasonable choice in the design and application of LCARFs.

**Figure 7:**
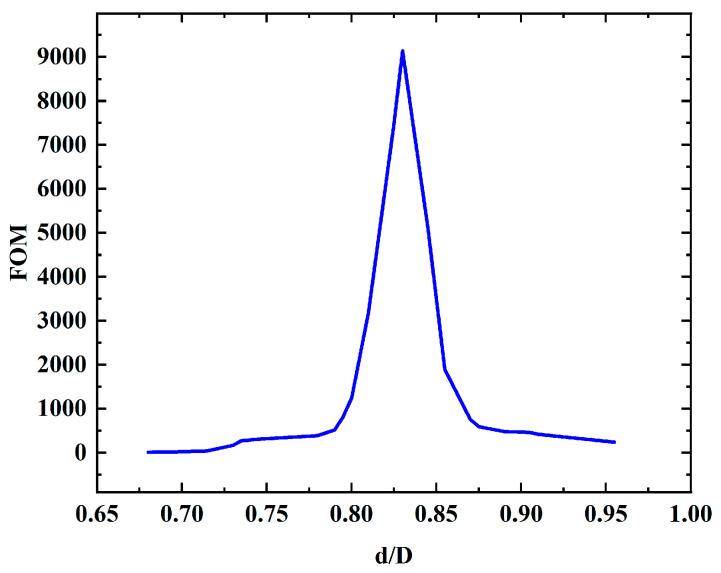
FOM at different d/D for six-tube structure.

To quantify the degree of HOM suppression, a figure of merit (FOM), FOM = α_11_/α_01,_ was introduced, where α_11_ and α_01_ denote the loss of the LP_11_ and LP_01_ core mode (both expressed in dB/m). The core diameter is fixed to be 30.5 μm, and the operating wavelength is set at 532 nm. The ratio of the diameter of the cladding tube and core (d/D) is modified by changing the diameter of the cladding tube, and Figure 7 shows FOM as a function of d/D. When d/D = 0.83, FOM reaches the maximum value of 9136, and it turns out that the effective RI of the HOM in the core and cladding tube mode match best at this point. Therefore, the high-order-mode energy in the core region is strongly released through resonance coupling and quickly leaks to the surrounding environment; hence, the best single-mode characteristic obtained.

**Figure 8:**
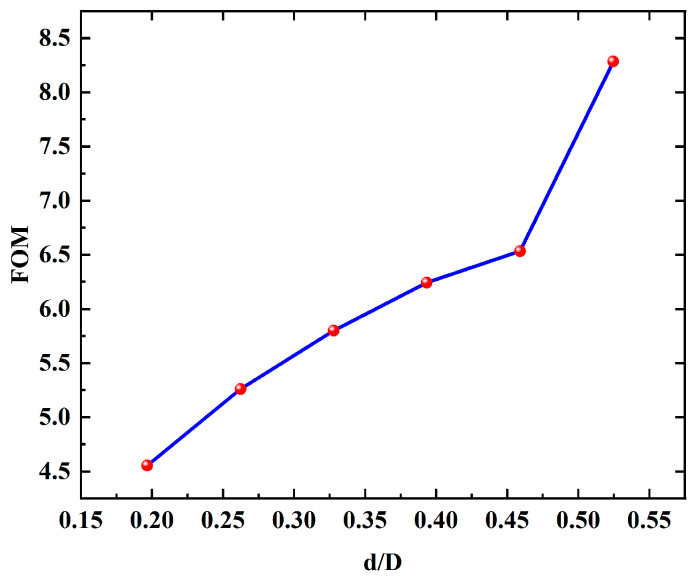
FOM at different d/D for eight-tube structure.

In addition, the change in FOM with d/D within the allowable range of the structure for the eight-tube structure was also discussed. As shown in Figure 8, in the range of d/D from 0.2 to 0.55 (the maximum value of d/D when cladding tubes do not overlap), FOM shows a monotonically increasing trend with an increase in d/D value. While the maximum value is less than 8.5, which is far less than the maximum value in the case of six-tube structure.

## 5. Conclusions

In this study, the confinement loss and single-mode characteristics of LCARFs at different filling conditions and geometrical parameters were analyzed theoretically. The numerical investigations revealed that the selectively liquid-filled ARF exhibits a lower confinement loss than the fully liquid-filled ARF. This selective filling method enhances the effective RI difference between the core mode and the cladding mode of the LCARF, thus avoiding the phase-matched condition and resulting in a lower confinement loss. Furthermore, the effect of geometrical parameters on the single-mode characteristics of LCARFs was investigated. By optimizing the capillary thickness and diameter ratio, an optimal single-mode transmission structure was achieved. Specifically, a structure with 500 nm-wall thickness, a six-tube cladding structure, and a diameter ratio (d/D) of 0.83 can provide the best single-mode characteristic. This advancement will establish a robust theoretical framework and provide valuable support for enhancing the LCARF applications in optofluidics.

## Figures and Tables

**Table 1:** Geometrical parameters of the investigated ARFs.

Core Diameter (D)	Capillary Thickness (t)	Capillary Inner Diameter (d)	Cladding Diameter
30.5 μm	1000 nm	12 μm	74.5 μm

## Data Availability

All the data presented in this study are available in this article.
